# Pan Traps for Tracking Honey Bee Activity-Density: A Case Study in Soybeans

**DOI:** 10.3390/insects11060366

**Published:** 2020-06-12

**Authors:** Ashley L. St. Clair, Adam G. Dolezal, Matthew E. O’Neal, Amy L. Toth

**Affiliations:** 1 Department of Ecology, Iowa State University, Evolution, and Organismal Biology, 251 Bessey Hall, Ames, IA 50011, USA; amytoth@iastate.edu; 2 Department of Entomology, University of Illinois Urbana-Champaign, 505 S. Goodwin Ave., Urbana, IL 61801, USA; adolezal@illinois.edu; 3 Department of Entomology, Iowa State University, 1344 ATRB 2213 Pammel Dr., Ames, IA 50011, USA; oneal@iastate.edu

**Keywords:** *Apis mellifera*, bee bowl, pan trap, moericke trap, activity-density, agriculture, foraging activity

## Abstract

To study how honey bees utilize forage resources and guide pollination management plans in crops, a multitude of methods have been developed, but most are time consuming, costly, and require specialized skills. Colored pan traps for monitoring activity-density are a simple, efficient, and cost-effective alternative; however, their usefulness for studying honey bees is not well described. We examined if trap color, location within a field, and the presence of managed colonies affected estimates of honey bee activity-density within soybean fields. Soybeans are visited by pollinators but do not require these visits for seed development. Pan traps, especially those colored blue, captured more honey bees when colonies were present. There were no differences in activity-density based on placement of traps within a field nor with increasing distance from colonies. Throughout the season, activity-density in soybeans was constant but tripled after soybean ceased blooming, suggesting spikes in pan trap captures may indicate periods of forage scarcity. Activity-density did not correlate with the population size of worker bees at a site, but did correlate with number of colonies present. We conclude that pan traps can be useful for assessing honey bee activity, particularly for estimating colony presence and identifying times of forage scarcity.

## 1. Introduction

The use of colored pan traps is a simple, efficient, and cost-effective technique to quantify insect communities [1,2,3]. Pan traps (or ‘bee-bowls’) can be a useful tool for monitoring bee communities [4,5,6,7]. However, there are drawbacks associated with this method that limit the ability of the traps to accurately quantify the community of bees in an area. It is widely suggested that pan traps are biased towards collections of certain families [8,9,10,11], although this may depend on the habitat and geographic region being sampled [10,12]. Furthermore, surrounding floral landscape variables may influence the activity-density of bees [13]. Consequently, it is suggested that researchers employ other methods, such as targeted sweep netting, to sample the bee community as an alternative or addition to pan traps [9,14]. Nonetheless, pan traps have a long history of use to estimate relative abundance and diversity of bees across treatments, landscape types, etc. [8,15]. Bee communities can arguably be better sampled directly from the flowers that they visit via netting [8,9]; however, this method also has limitations due to labor requirements and variability in researcher netting skills [16]. Pan traps provide an alternative that reduces these issues while successfully capturing many members of the pollinator guild [17]. 

Surprisingly, pan traps are rarely used to monitor managed honey bees (Hymenoptera: Apidae, *Apis mellifera* L.), and this species differs from many of the bees commonly found in pan traps because they are semi-domesticated, human managed, and live in large, eusocial colonies [18]. There are well established methods for measuring honey bee worker populations within colonies, foraging activity by individual colonies, as well as foraging activity in the field [19]. For this reason, honey bees can be a useful model for experimentally investigating the potential drawbacks of pan trapping listed above. 

In addition, managed honey bees across the US are experiencing worryingly high colony declines, especially in agricultural regions [20,21]. Research efforts have focused on estimating the occurrence of honey bees within a target crop field, information which is fundamental for pollination management and understanding how honey bees utilize foraging resources. By tracking honey bee activity-density in agricultural landscapes, we can estimate their response to variation in forage and possible exposure to pesticides [22,23,24]. In cropping systems that require pollination, honey bees are often placed next to a field in an attempt to increase pollination of the adjacent crop [25,26,27]. Quantifying honey bee activity within a crop field can help determine the relationship between the stocking rates of crops requiring pollination [19,28,29]. Current methods, such as observing honey bee waggle dances [30,31,32], radio frequency ID tagging [33,34], and direct observations of honey bee foraging [35,36] are limited in scope, time consuming, laborious, and can require highly specialized skills. Pan traps could be an efficient alternative, but it is unclear how well they estimate honey bee activity-density.

Honey bees are not as commonly reported in pan traps as other bee species, possibly due to traps being biased towards capturing certain species, namely small bees in the family Halictidae [9,37,38]. However, other factors related to foraging behavior patterns or plant and color preference, may explain an absence of honey bees in pan traps. Despite a potential bias, pan traps do capture honey bees, especially where honey bees are native, or where feral colonies are abundant [39,40,41]. Even when colored pan traps are deployed in crops that do not require insect-mediated pollination, honey bees have been reported as present, albeit in low numbers [42,43,44]. In regions where honey bees are not native or feral colonies are absent, few honey bees are captured, possibly because they are simply not present in the surrounding environment [45]. However, how pan traps are used (e.g., traps placed on the ground rather than elevated at flower height) can affect their ability to collect honey bees [9,46]. In general, pan traps estimate activity-density, i.e., the movement of an insect through a landscape coupled with its population density [47]. Assuming that honey bees are present, factors that affect the activity of a honey bee within a sampled area would affect the abundance of honey bees captured in a pan trap. 

Pan trapping has been identified as a method which captures the greatest activity-density of a pollinator community in agricultural fields compared to sampling methods used by applied entomologists to study insect pests of crops (e.g., yellow sticky traps and non-target sweep netting) [42,44,48]. Although these studies confirmed the presence of honey bees in crop fields using pan traps, they revealed a low level of honey bee activity-density, with honey bee foragers contributing a small percentage (0.005%) of the entire bee community captured [42,43,44]. However, it was not known whether honey bee colonies were present where these studies were conducted, let alone their population density. Additionally, these studies sampled for a limited period, potentially missing changes in flowering resources within or around the crop field, which are expected to affect the seasonal activity of honey bees.

Our goal was to investigate the usefulness of pan traps to monitor honey bee activity-density by evaluating the season-long activity-density of honey bees with colored pan traps in a crop field. Additionally, we used honey bees to experimentally test whether pan traps accurately represent bee abundance by altering the number of colonies present. We also explored how the floral landscape influences capture rate by comparing pan trap estimates of activity-density with variation in the surrounding land cover. We conducted this study in central Iowa, a region largely committed to the production of corn and soybean [49], with a homogenous landscape comprised of low floral diversity [50]. Managed honey bee colonies are kept within this region, and usually registered through an optional state registry; feral honey bee colonies are uncommon. Contributing to a lack of feral colonies is an absence of non-cropped features in the landscape [51], including forest, a primary nesting habitat for feral honey bees in temperate climates [52]. To confirm that honey bees were present, we placed colonies either adjacent to or within soybean fields in which pan traps were deployed, and monitored the population within the colonies throughout the growing season.

Soybean is a self-pollinated crop that does not require insect-mediated pollination; nonetheless, it is considered a moderately attractive crop for honey bees [53], and a potentially valuable, if not abundant source of nectar [54,55,56]. Furthermore, floral visits by honey bees has the potential to increase soybean yield [57,58,59]. We designed two experiments, and then conducted a comparative analysis of both, to answer eight fundamental questions about the utility of pan traps to measure honey bee activity-density (Table 1). In total, we conducted a large-scale assessment by sampling honey bees over a four-year period, within 44 soybean fields in central Iowa. 

## 2. Methods

### 2.1. Experiment One: Does Honey Bee Activity-Density Vary with Trap Color, the Presence of an Apiary, Bee Population within an Apiary, Surrounding Landscape Composition, Trap Placement, and Soybean Phenology?

Our first experiment explored if the activity-density of honey bees depended on trap color, the presence of colonies, trap placement, and how activity-density varied across the season (Table 1, questions 1–4). Furthermore, at sites where colonies were placed, we explored whether honey bee activity-density varied based on the population of capped brood (i.e., developing pupae) and adult bees throughout the season (Table 1, question 5) and whether the composition of the surrounding landscape influenced activity-density (Table 1, question 6). 

### 2.2. Site Selection

During the summers of 2015 and 2016, we identified 18 and 20 soybean fields in central Iowa, respectively. We used 22 commercial soybean fields managed by Iowa State University (ISU) and 16 fields managed by private farmers (10 ISU and 10 private farms for Apiary (+) sites). All fields were conventionally managed for bulk soybean production and 20 ha or greater in size. Fields were located at least 3.2 km from each other to ensure that measurements of honey bee activity-density were independent observations [32]. All soybeans were planted with a treated seed; ISU fields were planted with only a fungicide seed treatment (Fluopyram, ILeVO, Bayer, Pittsburgh, PA, USA), and private fields were planted with a treatment composed of an insecticide and fungicide (imidacloprid and ILeVo, respectively; Acceleron, Bayer, Pittsburgh PA, USA). Exposure to dust from seed-applied neonicotinoids (i.e. imidacloprid) at planting can affect honey bee navigation [60,61], potentially altering the results of our experiment; therefore, honey bee colonies were not placed at soybean fields until after the fields were planted and had reached the vegetative cotyledon stage of growth (VC) (see below). Weeds were managed with glyphosate and no insecticides were applied to soybean foliage or in fields directly surrounding them. The developmental growth stage of soybeans was evaluated to determine when flowers were present [62,63]. Growth stages in which flowers were present spanned the R1 (at least one open flower at any node on the main stem) to R4 (pods 2 cm at four uppermost nodes, flowers still present on main stem) stages.

### 2.3. Honey Bee Apiary Placement 

We placed apiaries at a subset of these soybean fields to determine if honey bee activity-density varied with the presence (designated as Apiary (+)) and absence (designated as Apiary (−)) of honey bees. Each apiary consisted of four colonies placed 3 m from a field edge. Of our 18 fields in 2015, we randomly selected 10 soybean fields to receive an apiary. Honey bees are most likely to encounter neonicotinoids from seed coatings during and directly after planting [64,65]. Therefore, in an attempt to reduce the possible effect of insecticides in our study, apiaries were transported to soybean fields at Apiary (+) sites on 6 June 2015 after 90% of the corn and soybean had been planted in Iowa [66] and all experimental fields had reached the VC reproductive growth stage. Prior to transporting apiaries to soybean fields, all colonies were standardized by size following methods from Dolezal et al. [56]. Apiaries remained at a soybean field edge throughout the season until 12 October 2015 when they were moved to a separate ISU research field for overwintering. To confirm that Apiary (−) sites did not have any other managed honey bee colonies present within 1.6 km of the field, we checked the state of Iowa’s voluntary registry for colonies (DriftWatch Inc., West Lafayette, IN; https://ia.driftwatch.org/map). In addition to the registry, we also scouted all fields directly neighboring our experimental field to confirm the absence of managed honey bee colonies. These efforts resulted in eight Apiary (−) locations. 

Due to the yearly crop rotation for the production of corn and soybean, we selected new fields each year. For the 20 new fields selected in 2016, we repeated the design used in 2015, selecting 10 soybean fields to receive an apiary and 10 fields that did not. Apiaries were transported to soybean fields on 22 May 2016 when 85% of corn and soybean were planted [67], and moved for overwintering on 18 October 2016. 

As part of a larger project, each of the colonies within an apiary placed at a field edge during 2015 and 2016 was inspected every other week following methods from Dolezal et al. [56] beginning on 24 June 2015 and 22 May 2016 and continued through 12 October 2015 and 18 October 2016. These inspections occurred on weeks alternating with pan trap estimates of activity-density. During each inspection, we measured capped brood population, and adult bee population (2016 only). To investigate how activity-density of honey bees in pan traps (see below) varied with the total number of bees at a site on a particular date, we compared population data across the season to the pan trap data at each site where colonies were placed. Furthermore, we quantified the landscape composition surrounding each field where honey bees were placed following methods from Dolezal et al. [56]. Landscape composition within 1.6 km was categorized into four land cover categories; cropland, grassland, woodland, and developed land. To investigate whether activity-density varied with surrounding landscape composition, we compared pan trap estimates to each land cover category.

### 2.4. Estimating Honey Bee Activity-Density

**Figure 1:**
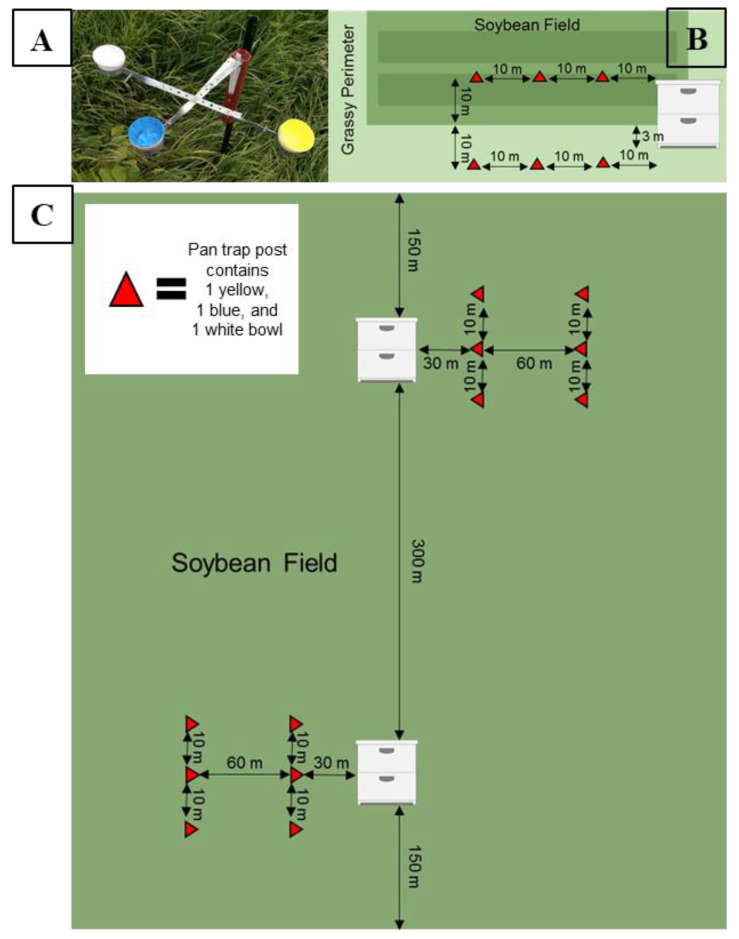
(**A**) Pan trap design for a single post constructed to hold three 3.2 oz bowls painted fluorescent yellow, fluorescent blue, or left white. (**B**) Experimental design of soybean fields in 2015 and 2016 (experiment one). Four colonies were placed 3 m from the field edge. In 2015, three posts were placed in the interior of the field adjacent to the colonies. In 2016, three posts were placed in the interior and the exterior of the field adjacent to the colonies. (**C**) Experimental design for soybean fields in 2017 and 2018 (experiment two). An apiary of 16 colonies was placed inside the fields (8 at each sub-site). In August, half of the colonies were removed from each sub-site, resulting in 4 colonies per sub-site remaining. Pan trap posts were placed 30 m and 90 m from the colonies at each sub-site.

To estimate honey bee activity-density, we used colored pan traps based on the design of Droege et al. [5] with modifications per Gill and O’Neal [42]. In 2015, traps were deployed on posts, such that each post held three 3.2 oz. bowls (Solo^®^ brand). We were interested in which of the common trap colors were more attractive to honey bees, so each post contained traps painted either fluorescent yellow, fluorescent blue, or left unpainted and white (Figure 1A). Each field had three posts with three traps of each color (nine traps total) placed 10 m apart and 10 m into a soybean field in a row that ran parallel to the field edge, with one end adjacent to honey bee colonies when present (Figure 1B). Traps were deployed for 24 h, every other week, on days with low cloud cover, no precipitation, and low to no wind (<10 mph). During each collection, the pan traps were adjusted on the post so that their height was level with the soybean plant canopy. Each trap was filled with a soap-water solution consisting of 3% Dawn^®^ dish soap and 97% water. We sampled bees for 13 weeks; 1 July through 24 September 2015. In 2016, we repeated the 2015 sampling design and added an additional three posts into the grassy perimeter of the field to test whether placement of the trap affected honey bee activity-density (Figure 1B). We sampled bees for 13 weeks; 15 June through 9 September in 2016. All estimates of honey bee activity-density were reported as honey bees per trap.

### 2.5. Statistical Analysis

All statistical analyses were performed in SAS 9.4. To explore which pan trap color was most attractive to honey bees, we performed a mixed model analysis of variance (PROC GLIMMIX) with trap color as the main effect and site-year as a random effect. For all analyses thereafter, all three trap colors were combined for a site such that bees per trap represents the total number of traps at a site. To investigate whether honey bee activity-density varied with the presence of honey bee colonies, we performed a t-test with pooled variance (PROC TTEST) comparing Apiary (+) sites to Apiary (-) sites. The sampling days were combined such that the analysis compares honey bees per trap per site. To explore whether activity-density of honey bees differed between pan traps 10 m inside the soybean field compared to traps placed 10 m in the exterior grassy perimeter, we performed a t-test (PROC TTEST). For this test, we used only data from the Apiary (+) sites in 2016 pooled across the season, such that the analysis compared honey bees per trap per site. Because we observed no significant difference between honey bee activity-density in the interior of soybeans compared to the grassy perimeter (see results below), we included trap collections from both locations at Apiary (+) sites in 2016 in the analysis. Honey bee activity-density was pooled across the season and standardized by trap number, such that the analysis compared honey bees per trap color per site. We used least squared comparison of means with Tukey adjustment to evaluate post hoc comparisons of trap colors. To examine how honey bee activity-density in soybean fields varied across the season and with soybean phenology, we performed a mixed model analysis of variance (PROC GLIMMIX) with date as a main effect and site-year as a random variable. For this analysis, we analyzed honey bees per trap per site for Apiary (+) sites only. Sampling dates took place on the same weeks across 2015 and 2016 with the exception of 15 June and 6 September, which was only sampled in 2016, and 24 September, which was only sampled in 2015. For those exceptions, the analysis only includes colonies from a single year (n = 20). All other dates in the analysis include colonies from both years (n = 40 colonies per date). We used least squared comparison of means with a Tukey adjustment to evaluate post hoc comparisons of sampling dates.

We investigated whether the total population of capped brood and adult bees at a site on specific dates correlated with the pan trap estimates of activity-density by performing a linear regression (PROC REG). Using data from 2015 and 2016 at Apiary (+) sites, with the exception of adult bee population, which was only measured in 2016, we compared the total honey bees in traps per site with the total population metric (sum of all four colonies) per site at each sampling date. Due to the inflated values of honey bee activity-density after soybean senescence (see results), we excluded the sampling date 24 September 2015 from the analysis. Because colony inspections and pan trap samples took place on alternating weeks, we created an estimate for capped brood and adult bee populations at each site on pan trap sampling dates by averaging the values from the colony inspection dates prior to and post pan trap assessment dates. This resulted in an extrapolated value of total population that was the midpoint between the two colony inspections and represents the expected population on the dates pan traps were sampled.

Using linear regression, we compared the proportion of each land cover category in the surrounding landscape with activity-density estimated at Apiary (+) sites in 2015 and 2016. Activity-density across the season, excluding 24 September 2015, was summed and standardized by trap number and is represented as honey bees per trap per site. The ability of a honey bee colony to successfully reproduce is not independent of the surrounding landscape, as honey bees require resources from the landscape (pollen and nectar) to rear new bees. Therefore, it is possible that colony population and surrounding land cover interact, and this interaction may affect the activity-density of honey bees in pan traps. To investigate whether activity-density of honey bees in soybeans varies with surrounding land cover and colony size, we performed a mixed model analysis of variance (PROC MIXED). We treated maximum capped brood or adult bee population at a site and proportion land cover as the fixed effects and siteyear as a random variable. This model was repeated for each land cover category and colony population interaction. We assumed that larger colony populations would be capable of utilizing more landscape surrounding the field; therefore, we calculated the maximum brood and adult bee population at a site and compared the total number of honey bees captured in pan traps at the sampling date equivalent to the maximum value of colony size.

### 2.6. Experiment Two: Does Honey Bee Activity-Density Vary with Distance from Colonies?

A trap’s distance from a colony may affect the abundance of bees collected. We compared if trap distance from a colony affects estimates of honey bee activity-density (Table 1, question 7). We also measured wing wear on bees captured in traps to determine whether traps capture experienced bees in the act of foraging or captured inexperienced bees conducting an orientation flight around the colony.

### 2.7. Site Selection

During the summer of 2017, we selected three soybean fields maintained by the ISU Research Farm that were 20 ha or greater and at least 3.2 km apart to measure honey bee activity-density. Two fields were planted with a soybean aphid resistant variety (IA2010-RA12, Rag1+Rag2), seeds were left untreated and no foliar insecticides were applied. Pre-emergent weeds were managed with pendimethalin (Prowl H_2_O, BASF, Florham Park, NJ, USA) and sulfentrazone (Sonic, Corteva Agriscience, Wilmington, DE, USA). Post-emergent weeds were managed with spot treatments using a backpack sprayer with clethodim (Clethodim 2E, Albaugh LLC, Ankeny, IA, USA), fomesafen (Flexstar, Syngenta, Wilmington, DE, USA), and pyroxasulfone (Zidua, BASF). A third field was planted with an insecticidal seed treatment (imidacloprid, Pioneer Premium, Johnston, IA, USA) and when the field was in full bloom (reproductive stage R3), it was sprayed with a foliar application of Warrior II (lambda cyhalothrin, Syngenta) on 25 July 2017. In this field, post-emergent weeds were managed with glyphosate (Roundup PowerMAX, Bayer Crop Science, Research Triangle Park, NC, USA). Growth stage of soybeans was monitored weekly from June through September.

In 2018, we used the same guidelines to select soybean fields in 2017, adding three new soybean fields. Two fields were planted with a soybean aphid resistant variety (IA2010-RA12, Rag1+Rag2) with no insecticide or fungicide applied to the seed and no applications of insecticides to foliage. Weeds were managed as described above. A third field was planted with an insecticidal seed treatment (imidacloprid, Pioneer Premium) and sprayed with a foliar application of Warrior II (Syngenta) on 17 July 2018 when the field reached the R3 growth stage. Soybean growth stage was assessed weekly from June through September. 

### 2.8. Honey Bee Apiary Placement 

In 2017, each soybean field received an apiary of 16 colonies on 2 June after the fields were planted and post-emergence herbicides were applied. To ensure honey bees would forage within the crop, colonies were placed in the interior of soybean fields rather than at the field edge. Within a field, apiaries were placed at two sub-sites such that each sub-site consisted of eight colonies, to reduce bees drifting between colonies. Sub-sites were at least 150 m from the closest field edge and 300 m from the adjacent sub-site (Figure 1C). All colonies remained in the soybean fields until 10 August 2017. At that time, half of the colonies from each sub-site (n = 8) were moved out of the field to another location as part of a separate experiment. The remaining colonies (n = 8) stayed in the soybean fields. After the experiment, all colonies from apiaries were moved to an ISU research field for overwintering on 12 October 2017. In 2018, the same procedure from 2017 was repeated, with apiaries placed in soybean fields on 8 June 2018 and a subset moved out on 9 August 2018. Colonies were moved to overwinter at the ISU research apiary on 13 October 2018.

### 2.9. Estimating Honey Bee Activity-Density

During 2017 and 2018, honey bee activity-density was measured using the same pan trap design as described above in experiment one. The placement of the pan traps varied, with six posts at each of two sub-sites within a soybean field (12 posts per field, 6 posts per sub-site). Each post had three pan traps of either blue, yellow, or white, resulting in a total of 36 pan traps per field and 18 pan traps per sub-site. To evaluate whether activity-density of honey bees decreased with increasing distance from an apiary, three posts, each with three pan traps were placed at two distances from the apiary (30 m and 90 m) of each soybean sub-site (Figure 1C). At each distance, individual posts were 10 m away from the next closest post along a straight line. Each soybean field was considered the experimental unit, and sub-sites were not far enough in distance to be an independent sample, we summed all of the honey bees captured across sub-sites resulting in one 30 m and one 90 m activity-density estimate per field. We standardized honey bee activity-density by trap so that estimates at each distance are represented as honey bees per trap per site. We sampled activity-density every other week from 16 June through 6 September 2017 and from 26 June through 18 September 2018. 

### 2.10. Assessing Honey Bee Wing Wear

We quantified honey bee wing wear to estimate flight experience of bees captured in the pan traps. To measure wing wear, we clipped both forewings off specimens captured in pan traps located at a 30 m and 90 m distance from colonies in soybeans across the season. Forewings were taped onto a slide under a coverslip and evaluated by microscopy on a scale of 1–6 following modified methods by Mueller and Wolf-Mueller (Figure S1) [68]. Wing wear of both the left and right wing were averaged producing one measure of wing wear per specimen.

### 2.11. Statistical Analyses

All statistical analyses were performed in SAS 9.4. To examine whether or not honey bee activity-density varied with distance from the apiary (30 m or 90 m), we performed a mixed model analysis of variance (PROC GLIMMIX) with trap distance, collection date, and whether or not the field was sprayed with an insecticide as the main effects and siteyear as a random factor. Because our sampling was based on the soybean crop phenology rather than Julian date, each sampling date in 2017 corresponded to the same crop phenology period sampled in 2018. Therefore, we summed data across the two years. We performed post hoc comparisons of least squared means with Tukey adjustments to compare differences between dates and trap distance. 

To investigate whether honey bees in pan traps were foragers, we performed a one-tailed t-test (PROC TTEST) to determine if wing wear of bees in traps was significantly different from zero. A separate t-test was performed on bees collected in traps at a 30 m and 90 m distance from the apiary. To determine whether wing wear of bees collected at a 30 m and 90 m distance from the apiary differed, we conducted a mixed model analysis of variance with distance from the apiary as the fixed effect and siteyear as the random factor. For all tests of wing wear, each bee captured in the trap was considered an experimental unit, rather than the field.

### 2.12. Combined Analysis: Does Honey Bee Activity-Density in Soybeans Vary with the Number of Colonies Present?

Lastly, we conducted an exploratory analysis that combined pan trap data over four years from multiple soybean fields in which the number of colonies adjacent to or within them varied. In this way, we could investigate if variation in the activity-density of honey bees in a field is explained by the number of colonies nearby (Table 1, question 8). 

To investigate the potential relationship between the number of honey bee colonies present and the amount of honey bees captured in pan traps, we combined data from the previous two experiments. In Experiment one; we had 18 sites with no colonies and 20 sites with 4 colonies adjacent to a soybean field. In Experiment two, we had six sites with 16 colonies present within soybean fields between June and August, and 8 colonies present in those same fields after August. Based on the results from experiment one, we concluded honey bee activity-density did not vary prior to and during soybean bloom (May to early September). Therefore, the combined analysis included all data from May through early September, excluding only 24 September 2015. During 24 September 2015, soybean plants had ceased blooming and the activity-density likely did not represent foraging on soybean flowers (see results in experiment one for an explanation). Based on results from experiment two, we observed no difference in the activity-density of honey bees between the two distances from the colony (see results below), therefore, data from the two distances was combined. Due to the unequal number of pan traps in experiment one compared to experiment two, all pan trap data in the analysis is represented as the number of honey bees per trap per site. Because there were a different number of dates sampled between the two experiments and within experiment two a different number of sampling dates represented for each density of colonies (16 colonies in June-August, 8 colonies in August-September), we further standardized the activity-density of honey bees by the number of days sampled at the respective densities. Thus, for the combined analysis, honey bee activity-density is estimated as the number of honey bees per trap per site per day. After all the data were standardized, we performed a linear regression analysis (PROC REG) in SAS.

## 3. Results

### 3.1. Experiment One: Does Honey Bee Activity-Density Vary with Trap Color, the Presence of an Apiary, Bee Population within an Apiary, Surrounding Landscape Composition, Trap Placement, and Soybean Phenology?

Over 2015 and 2016, we collected a total of 294 honey bees in pan traps, with 124 and 170 collected in 2015 and 2016 respectively. In addition to honey bees, the pan traps also collected other native bee species that were identified as part of a larger study. Of the >50 bee species collected within pan traps, honey bees were the fifth most abundantly collected species, suggesting that pan traps have the ability to collect honey bees as efficiently as other native species. 

**Figure 2:**
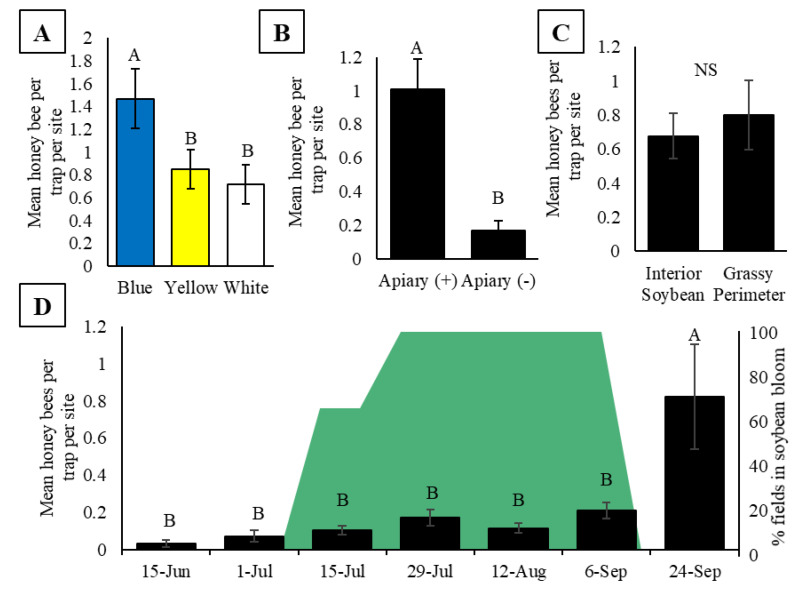
(**A**) Mean honey bee activity-density (bees per trap per site) in pan traps inside soybean fields during 2015 and 2016 in central Iowa. (**B**) Mean honey bee activity-density at Apiary (+) soybean fields with pan traps located 10 m inside soybean fields compared to traps placed 10 m into the grassy perimeter of the field during 2016 in central Iowa. (**C**) Mean honey bee activity-density in soybean fields in central Iowa during 2015 and 2016 by trap color. (**D**) Mean seasonal honey bee activity-density inside Apiary (+) soybean fields (black bars) during 2015 and 2016. The green block represents the percentage of soybean fields in bloom (R1-R4) at each sampling week. For A-D, activity-density is reported as the mean ± one standard error of honey bees captured in pan traps per site. Different letters signify *p* < 0.05 for post hoc comparison of least squared means.

The number of bees in the pan traps differed significantly between colors (F_2, 38.05_ = 8.44, *p* = 0.0009; Figure 2A) with blue traps capturing more honey bees than both yellow and white traps (Figure 2A). Because the experiments outlined within this study were part of larger experiments also assessing the wild bee community, we continued to use blue, yellow, and white pan traps despite finding that blue traps capture more honey bees. All results from this point forward reflect collections from all three colors combined. 

In total, 245 honey bees were collected in pan traps at Apiary (+) sites, compared to the 45 honey bees collected at Apiary (-) sites, resulting in significantly higher activity-density of honey bees at Apiary (+) compared to Apiary (-) soybean fields (T_36_ = 4.33, *p* = 0.0001; Figure 2B). At Apiary (+) fields, there was no observed difference in activity-density between traps placed 10 m inside the soybean field compared to those placed 10 m inside the grassy perimeter of the field (T_18_ = 0.51, *p* = 0.62; Figure 2C). Activity-density varied by date (F_6, 86.88_ = 9.32, *p* ≤ 0.0001), with significantly more bees captured in traps on 24 September (after soybeans ceased blooming) compared to all other dates including before and during soybean bloom (Figure 2D, Table S1). 

**Figure 3:**
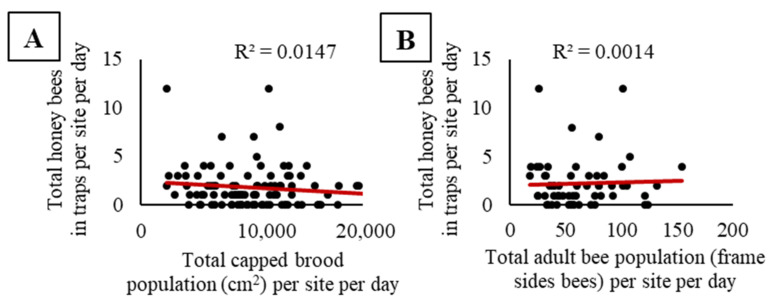
No significant relationships were found between honey bee activity-density (bees per trap per site per day) in pan traps with (**A**) total capped brood population at a site nor (**B**) total adult bee population at a site for each sampling day. Capped brood populations are from 2015 and 2016, while adult bee population was only measured in 2016.

**Figure 4:**
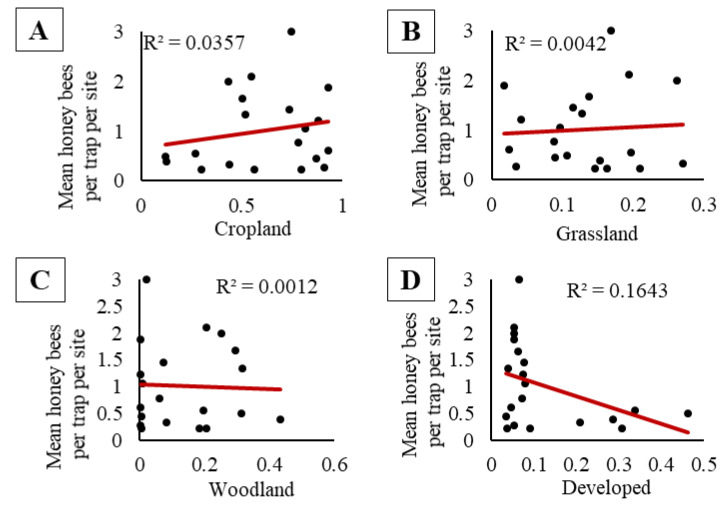
No significant relationships were found between honey bee activity-density (bees per trap per site) in pan traps with proportion of (**A**) cropland, (**B**) grassland, (**C**) woodland, nor (**D**) developed land cover within 1.6 km of the apiary at the field edge of soybean sites in 2015 and 2016.

We did not observe a significant relationship between the pooled activity-density of honey bees and pooled capped brood population (F_1, 98_ = 1.46, *p* = 0.23; Figure 3A), nor pooled adult bee population (F_1, 58_ = 0.08, *p* = 0.78; Figure 3B). At sites where honey bees were placed, there were no significant relationships between the activity-density of honey bees and the proportion of cropland, grassland, woodland, or developed land in the surrounding landscape (cropland, F_1, 18_ = 0.67, *p* = 0.42, Figure 4A; grassland, F_1, 18_ = 0.08, *p* = 0.79, Figure 4B; woodland, F_1, 18_ = 0.02, *p* = 0.89, Figure 4C; and developed land, F_1, 18_ = 3.54, *p* = 0.08 Figure 4D). When we compared the maximum capped brood and adult bee populations at a site combined with the proportion land cover, we observed no significant effects of apiary size (capped brood or adult bee population), no significant effects of land cover proportions (cropland, grassland, woodland, or developed), and no interactions on honey bee activity-density (Table S2). 

### 3.2. Experiment Two: Does Honey Bee Activity-Density Vary with Distance from Colonies and by Site Type?

**Figure 5:**
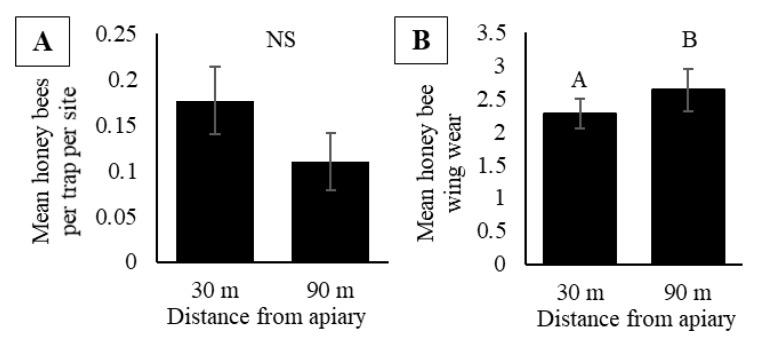
(**A**) Mean honey bee activity-density (bees per trap per site) in pan traps in soybean fields with increasing distance from the apiary (30 m or 90 m) in central Iowa during 2017 and 2018. (**B**) Mean honey bee wing wear of honey bees collected in pan traps placed 30 m and 90 m from the apiary. Results based on a mixed model analysis of variance. Data represent mean ± one standard error of the mean. Letters signify significant differences (*p* < 0.05).

Over 2017 and 2018, we collected a total of 184 honey bees in pan traps, with 65 and 119 collected in 2017 and 2018, respectively. At a 30 m trap distance from colonies, we collected 110 honey bees and 74 honey bees were collected at a trap distance of 90 m. There were no significant differences in honey bee activity-density based on trap distance from the apiary (F_1, 44_ = 2.44, *p* = 0.13), application of insecticide (F_1, 4_ = 0.14, *p* = 0.72) and activity-density did not vary by date (F_5, 44_ = 1.68, *p* = 0.16) (Figure 5A). There were no interactions between date and trap distance (F_5, 44_ = 2.22, *p* = 0.07), date and insecticide application (F_5, 44_ = 0.61, *p* = 0.70), nor between date, trap distance, and insecticide application (F_5, 44_ = 0.71, *p* = 0.62). Honey bees captured in pan traps had wings that were significantly more worn at a distance 90 m from the apiary compared to 30 m from the apiary (F_1, 175.4_ = 3.98, *p* = 0.05; Figure 5B). Wing wear at both distances was significantly greater than zero, indicating bees collected at both distances had been actively foraging before being captured (T_118_ = 10.24, *p* ≤ 0.0001 for 30 m and T_60_ = 8.35, *p* ≤ 0.0001 for 90 m).

### 3.3. Combined Analysis: Does Honey Bee Activity-Density in Soybeans Vary with the Number of Colonies Present?

**Figure 6:**
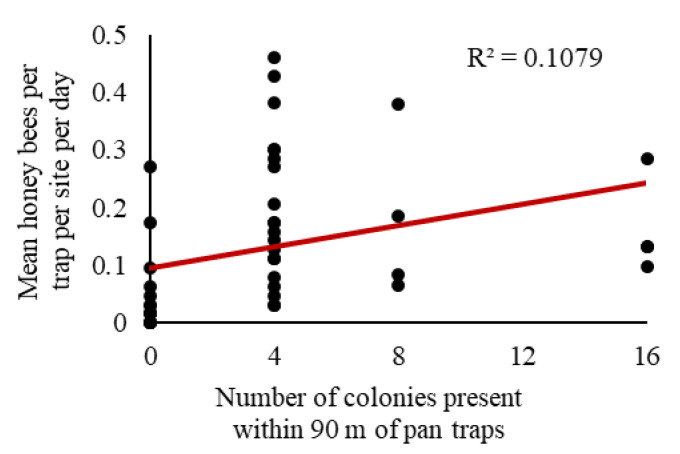
Correlation of colony number (0, 4, 8, or 16 colonies) with observed honey bee activity-density per trap per soybean site per day in fields in central Iowa from 2015 to 2018. Linear equation: y = 0.0092x + 0.0957.

We observed a significant relationship between honey bee activity-density and the number of colonies (F_1, 44_ = 5.32, *p* = 0.03); however, only 10.8% of the variation in activity-density was explained by the number of colonies present (r^2^ = 0.1079; Figure 6).

## 4. Discussion

Our results suggest that, in regions such as Iowa where honey bees are not native and feral colonies are uncommon, pan traps can be used to approximate honey bee activity when colonies are placed next to or within a focal crop field (Figure 2B). The use of multiple colors (blue, yellow, and white) are usually recommended to capture a diverse community of bee species [15,17,69]. Consistent with other studies suggesting honey bees have an innate preference for blue flowers [70,71], we found blue traps captured the most honey bees (Figure 2A), more than twice that of both yellow and white traps, and may be the best option for studies specifically targeting honey bee activity-density. The use of all three-trap colors throughout our study may have contributed to the low estimates of abundance of honey bees based on a per trap per site measurement. For honey bees captured in blue bowls, our collections of 1.5 honey bees per trap per site are consistent with collections of other common bee species captured in pan traps in this region [42,43,44,72,73]. 

The placement of a pan trap can affect how many pollinators are captured [5]. Unlike many wild bees, which forage 500 m or less from their nest location [74,75], honey bees are capable of longer foraging distances (>10 km) [76], and in agricultural landscapes commonly forage within 2 km of their colony [30,31,32]. As a result, it is necessary to place pan traps at multiple positions and distances from the colonies in order to best estimate how pan trap placement and honey bee activity-density are related. One caveat to our results from experiment one is that our pan traps were placed only 10 m from the colonies. At this distance, the placement of pan traps within the field or within the grassy perimeter adjacent to the field did not affect the estimate of activity-density when colonies were present at a field edge (Figure 2C). It is possible that traps placed at a 10 m distance may have captured not only foraging bees, but also younger, non-foragers in the process of orienting to the location of their colony [77]. 

To determine if traps placed further away gave a different picture, we compared the activity-densities in traps at 30 m and 90 m from colonies. We did not observe a difference in activity-density between these distances (Figure 5A), and the values were comparable to those at a 10 m distance. These observations suggest that when honey bee colonies are present near or within a large crop field (>20 ha), the bees collected represent the activity of foragers rather than orienting bees. The measurements of wing wear further support this conclusion. As honey bees make foraging flights to and from the colony their wings receive wear, related to the effort and experience spent foraging [78,79]. If only bees on orientation flights were captured in pan traps, we would expect very little to no wing wear as they would have left the colony only recently. Although we did occasionally capture bees with no wing wear, we observed that honey bees captured at distances 30 m and 90 m from the apiary had wing wear that was significantly greater than zero, suggesting that the traps captured experienced foragers (Figure 5B). Furthermore, we observed a greater wear in wings of honey bees at 90 m compared to a 30 m distance (Figure 5B), suggesting more experienced foragers may forage greater distances [80].

Because soybeans are grown in fields with dimensions exceeding 90 m in length, our results do not allow us to conclude that foraging takes place across the entire field. Reduced activity is likely to occur at distances greater than 90 m from an apiary. An additional caveat is the reduced replication of soybean sites in experiment two, which may have failed to capture enough variation in trap distance to see a significant effect of distance on activity-density. Further studies should aim to tease apart how honey bee activity-density estimates vary with trap distance from an apiary in and around fields by examining multiple distances with increased site replication.

We expected to see honey bee activity-density inside soybean fields change throughout the season, with the highest activity correlated with the peak bloom of soybean flowers, a resource which has been identified as valuable to honey bees in corn/soybean cropping systems [56]. Contrary to our expectations, we did not observe any variation in activity-density in soybeans prior to or during soybean bloom (Figure 2D). Prior to soybean bloom, it is likely that there was an abundance of alternative floral attractants (e.g., clover and tree pollen). However, strikingly, in late September 2015 (24 September), we observed honey bee activity-density in soybeans nearly tripled (Figure 2D). In late September, soybeans in Iowa have typically senesced to the point of no longer having leaves. The high density of honey bees observed in a soybean field at that time could be due to the pan traps being perceived as the only ‘flowers’ available, increasing their relative attractiveness to the bees at a time when no other flowers were present. This explanation corroborates suggestions that pan traps are biased towards collecting more bees when floral diversity and abundance are low [37,45,69]. Although the increased activity may be an overestimate of actual foraging effort, these data may still serve as a valuable indicator of honey bee foraging behavior by providing information on attraction of foragers to false signals when true floral resources are lacking. Peaks in captures of honey bees in pan traps may provide useful information about when honey bees face forage limitations in crops and other field types and can be a precursor for identifying when honey bee colonies may need to be moved to landscapes with more resources or provided supplemental feed by beekeepers. Additional studies are necessary to parse out the effects of forage limitation on bee activity-density. 

An alternative explanation for the peak in activity-density in late September could be a putative relationship between trap captures and honey bee population size. Honey bee foraging effort is proportionate to the needs of developing bees and increases with colony population size [81], particularly when brood development in the colony is high [82]. We would expect that as colony populations’ increase, so to would the activity-density of foragers within an adjacent field. To investigate the relationship between population of bees near a field and activity-density within the field more deeply, we compared pan trap estimates with the total capped brood bee and adult bee populations at a site on each sampling day. Results from this analysis revealed no relationship between the population of brood or adult bees near a site and the activity-density of bees within soybeans (Figure 3A,B), thus rejecting the idea that the September peak in honey bee activity-density is due to a peak in the worker bee population.

Unlike most solitary bees caught in pan traps, which forage independent of conspecifics [83,84], honey bee foraging behavior is influenced by the recruitment by scouts of additional foragers to a resource [85,86,87]. Once back at the hive, these scout bees communicate the location of the resource patch to recruit more foragers to exploit the resource [88,89]. Because the colored pan traps imitate a potential floral resource [90], they are likely to attract scout honey bees; however, if scouts are killed in traps, they will not recruit additional foragers to that location. For this reason, the recruitment-based foraging of honey bees may severely limit the ability of pan traps to accurately estimate the true activity-density of honey bees on a specific crop within a given area. Although we were not able to provide clarity to the issue of how pan traps and abundance of bees are related, the pan trap estimates do still provide valuable information about the general activity of honey bees in an area.

Although we did not see a relationship between the number of forager bees captured in the traps with the estimate of total number of bees in the apiary, pan traps may provide a rough estimate of the number of honey bee colonies. In our exploratory post-hoc analysis combining data across two experiments, we observed a significant positive association between honey bee activity-density and the number of colonies present (Figure 6), but the relationship was not particularly strong. There are several factors that could contribute to the weakness of this relationship, including low replication (e.g., for sites with eight and 16 colonies present), the attractiveness of soybeans to honey bees [53], and the attractiveness of other sources of forage within the surrounding landscape. The presence of bees within a crop field is likely related to the attractiveness of the crop as well as the resource availability in the surrounding landscape. Soybeans do not require insect pollination [91] and may not be as attractive as other plants commonly found in central Iowa (e.g., clover) [53,56,92]. We predict stronger relationships between the number of honey bees captured in pan traps with increasing number of colonies for crops that are more attractive to honey bees (e.g., canola).

Overall, our results provide insight regarding the usefulness of pan traps as a method of quantifying honey bee activity-density. Increases in crop production and the demand for honey bee pollination services [93] are occurring concurrent to widespread declines in managed honey bee colonies [20,94,95]. Thus, there is a need for improved methods to gain further insights into the effects agricultural systems have on honey bees and to gain a better understanding of how bees utilize different agricultural areas. 

It is important to note that although pan traps gave estimates of activity-density, they are not synonymous with foraging activity. While some studies have used pan trap collected bees to assess foraging resources by identifying the pollen on individual bees [42], we found this unlikely to be effective for honey bees as pollen pellets were usually washed off in the traps. If used in conjunction with direct observations, pan traps could broaden the scope of understanding of where bees forage in a landscape. 

## 5. Conclusions

We determined that pan traps, especially those colored blue, are a useful tool for assessing honey bee activity and colony density in soybeans within 90 m of an apiary. Pan traps create the ability to understand when bees are active in crop fields, information that can be valuable in addressing the potential for insecticide exposure in risk assessments. For example, in soybeans, we observed steady activity of honey bees throughout bloom (Figure 2D), a time when insecticides are likely to be applied for pest management [96]. Other applications include identifying the presence of nearby honey bee colonies in studies wishing to experimentally control honey bee presence, and choosing landscape conservation enhancements that target critical resource gaps for bees. Pan traps can provide activity estimates that can elucidate forager choices to utilize pollinator-dependent crops as opposed to crops in the surrounding landscape, which can be useful in determining stocking rates for various crops. Additional applications include studies that control honey bee presence for assessments of crop yields, wild bee activity in agricultural and conservation landscapes, and potential for pathogen spillover and competition between honey bees and wild bees. Our data also suggest restrictions associated with pan traps; at times when floral abundance and diversity are low, pan-traps can lead to inflated estimations of activity-density. 

## Figures and Tables

**Table 1:** Hypotheses related to the usefulness of pan traps as a method to estimate activity-density of honey bees in soybean fields.

Question #	Pan Trap Research Objective	Figure	Result ^a^
1	Does activity-density vary with pan trap color?	2A	✔
2	Does activity-density vary with colony presence/absence?	2B	✘
3	Does activity-density vary with placement in the interior vs. exterior of crop field?	2C	✔
4	Does activity-density vary across the season?	2D	✔/✘
5	When colonies are present, does activity-density correlate with bee population?	3	✘
6	Does activity-density vary with surrounding landscape composition?	4	✘
7	Does activity-density vary with distance from colonies?	5	✘
8	Does activity-density vary with number of colonies present?	6	✔

✔ represents a significant difference in activity-density occurred based on the result in the figure, ✘ indicates that no significant difference occurred. ✔/✘ indicates that activity-density significantly differed for some factors but not all factors ^a^.

## Data Availability

All data and related software code will be made publicly available upon publication.

## Supplementary Materials

The following are available online at https://www.mdpi.com/2075-4450/11/6/366/s1, Figure S1: Honey bee wing wear rating scale, Table S1: Means comparisons of activity-density across the season, Table S2: Maximum colony populations and landscape effects of activity-density.
