# Supplementary material for: Pan Traps for Tracking Honey Bee Activity-Density: A Case Study in Soybeans
Source: Insects. 2020 Jun 12;11(6):366. doi: 10.3390/insects11060366 (PMC7348912; doi:10.3390/insects11060366)
Supplement: Supplementary file 1 [file insects-11-00366-s001.pdf]

SUPPLEMENTAL INFORMATION

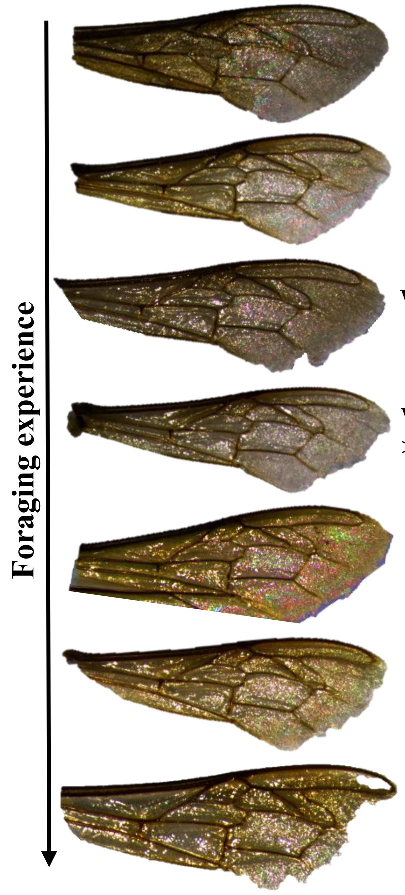

WW0: Wing margin (WM) intact

WW1: WM with 1-2 nicks

WW2: WM with 3-10 nicks

WW3: WM almost completely serrated with >10 nicks, but at least some WM intact

WW4: WM completely serrated with excisions less than half the width of the distal submarginal cell. No original WM intact

WW5: WM completely serrated with excisions more than half, but less than the entire width of the distal submarginal cell

WW6: WM showing major excisions greater than the width of the distal submarginal cell. Excisions may reach the distal crossveins

Figure S1. Honey bee wing wear rating scale as modified from Mueller and Wolf-Mueller 1993

Table S1. Least square means comparisons with Tukey adjustment of honey bee activity-density in pan traps across the season in soybean fields in central Iowa during 2015 and 2016.

Date	Date	Estimate	SE	DF	t Value	P value
1-Jul	12-Aug	-0.4000	0.8262	81.94	-0.48	0.6296
1-Jul	15-Jul	-0.3000	0.8262	81.94	-0.36	0.7175
1-Jul	15-Jun	0.3446	1.0150	91.93	0.34	0.7350
1-Jul	24-Sep	-6.7446	1.0150	91.93	-6.65	**<.0001**
1-Jul	29-Jul	-0.9000	0.8262	81.94	-1.09	0.2792
1-Jul	6-Sep	-1.2554	1.0150	91.93	-1.24	0.2193
12-Aug	15-Jul	0.1000	0.8262	81.94	0.12	0.9040
12-Aug	15-Jun	0.7446	1.0150	91.93	0.73	0.4651
12-Aug	24-Sep	-6.3446	1.0150	91.93	-6.25	**<.0001**
12-Aug	29-Jul	-0.5000	0.8262	81.94	-0.61	0.5467
12-Aug	6-Sep	-0.8554	1.0150	91.93	-0.84	0.4015
15-Jul	15-Jun	0.6446	1.0150	91.93	0.64	0.5270
15-Jul	24-Sep	-6.4446	1.0150	91.93	-6.35	**<.0001**
15-Jul	29-Jul	-0.6000	0.8262	81.94	-0.73	0.4698
15-Jul	6-Sep	-0.9554	1.0150	91.93	-0.94	0.3490
15-Jun	24-Sep	-7.0891	1.1790	102.9	-6.01	**<.0001**
15-Jun	29-Jul	-1.2446	1.0150	91.93	-1.23	0.2233
15-Jun	6-Sep	-1.6000	1.1685	81.94	-1.37	0.1746
24-Sep	29-Jul	5.8446	1.0150	91.93	5.76	**<.0001**
24-Sep	6-Sep	5.4891	1.1790	102.9	4.66	**<.0001**
29-Jul	6-Sep	-0.3554	1.0150	91.93	-0.35	0.7270

Table S2. Test of fixed effects investigating whether honey bee activity-density is affected by the interaction between the maximum brood or maximum adult bee population at a site and the surrounding land cover within 1.6 km.

	Capped brood population				Adult bee population			
	Effect	DF	F Value	Pr > F	Effect	DF	F Value	Pr > F
**Developed**	***Brood pop***	1, 16	1.94	0.182	***Bee population***	1, 6	0.99	0.358
	Developed	1, 16	3.23	0.091	***Developed***	1, 6	0.64	0.456
	Pop*Developed	1, 16	3.03	0.101	***Pop*Developed***	1, 6	0.45	0.526
**Cropland**	***Brood pop***	1, 16	0.14	0.716	***Bee population***	1, 6	0.92	0.374
	Cropland	1, 16	0.23	0.641	***Cropland***	1, 6	0.91	0.378
	Pop*Cropland	1, 16	0.15	0.704	***Pop*Cropland***	1, 6	1.12	0.33
**Woodland**	***Brood pop***	1, 16	0.2	0.664	***Bee population***	1, 6	0	0.962
	Woodland	1, 16	0.28	0.603	***Woodland***	1, 6	0.18	0.687
	Pop*Woodland	1, 16	0.29	0.596	***Pop*Woodland***	1, 6	0.87	0.387
**Grassland**	***Brood pop***	1, 16	0.55	0.469	***Bee population***	1, 6	0.35	0.578
	Grassland	1, 16	1.07	0.316	***Grassland***	1, 6	0.24	0.643
	Pop*Grassland	1, 16	0.74	0.404	***Pop*Grassland***	1, 6	0.39	0.556
